# Commentary on NNT Mediates Redox-Dependent Pigmentation *via* a UVB-And MITF-Independent Mechanism

**Published:** 2021-09-20

**Authors:** Jennifer Allouche, Inbal Rachmin, David E Fisher, Elisabeth Roider

**Affiliations:** 1 Department of Dermatology, Cutaneous Biology Research Center, Massachusetts General Hospital, Harvard Medical School, Charlestown, MA 02129, USA; 2 Department of Dermatology, Massachusetts General Hospital Cancer Center, Harvard Medical School, Boston, MA 02114, USA; 3 Department of Dermatology, University Hospital of Basel, 4031 Basel, Switzerland; 4 Department of Dermatology and Allergology, University of Szeged, 6720 Szeged, Hungary

## DESCRIPTION

Skin pigmentation is essential to balance skin cancer risk and vitamin-D production. The diversity of skin pigmentation is a result of the production, together with the type of the melanin, eumelanin (brown/black color) and pheomelanin (yellow/red color).Ultraviolet (UV) radiation as well as expression of different genes balance skin color *via* regulating pheo and eumelanin levels.

**Figure 1:**
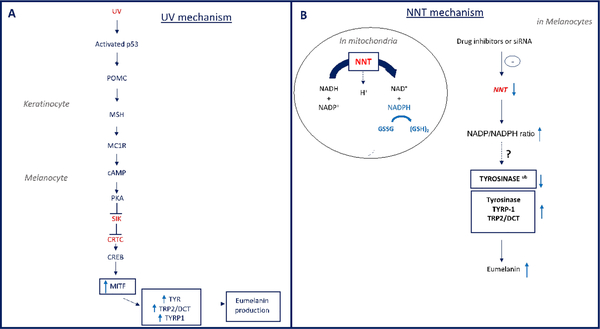
Skin pigmentation mechanisms.

1. In the established classic UV-cAMP-MITF-dependent “tanning pathway”, UV radiation mediates an increase in the transcription factor p53 in human keratinocytes, thereby stimulating the induction of Pro-Opiomelanocortin (POMC) and secretion of the Alpha Melanocyte-Stimulating Hormone (α-MSH). In turn MSH binds the Melanocortin 1 Receptor (MC1R) on melanocytes, which increases the expression of the Microphthalmia Associated Transcription Factor (MITF) directly regulating the transcription of Tyrosinase (TYR), Tyrosinase-Related Protein 1 and 2 (TRP1 and TRP2) and other pigmentation genes 1 (Figure 1A). Although pigmentation regulation by the MITF dependent pathway is well established, other pathways that are independent of MITF are not yet fully elucidated. Deciphering other melanogenesis mechanisms will facilitate understanding of pathogenesis associated with pigmentation disorders and the development of potential therapeutic options. Particularly, the question of how redox in mitochondria can affect pigmentation has not been reported. Recently, our group revealed a conserved mechanism of skin pigmentation which demonstrates an interplay between melanin and redox metabolism.
2. In our study entitled “NNT mediates redox dependent pigmentation *via* a UV and MITF independent mechanism”, Nicotinamide Nucleotide Transhydrogenase (NNT), a known regulator of mitochondrial redox levels expressed in the inner mitochondrial membrane was seen to mediate redox changes that impacted skin pigmentation as shown by *in vitro, ex vivo and in vivo* models as well as GWAS data analysis*. In-vitro*, inhibition of NNT using siRNA or small molecule inhibitors increased pigmentation in primary melanocyte cells as well as in melanoma cell lines. Interestingly the increase in pigmentation was only attributed to NNT inhibition since silencing other enzymes with similar enzymatic functions did not alter pigmentation, emphasizing the critical role of NNT in human pigmentation. The importance of NNT as a key regulator of pigmentation was further validated *in-vivo*, wherein deletion of NNT in both mice and Zebrafish, was associated with a significant increase of melanin towards the eumelanin levels. Finally, topical small-molecule inhibitors yielded human skin darkening. At a functional level, the increase of melanin was not mediated by the conventional MITF transcriptional regulation, but instead the effect of oxidative stress appeared to act on the stabilization of key enzymes involved in melanin synthesis, and which was accompanied by an increase in melanosome maturation, highlighting a distinct pigmentation mechanism (Figure 1B). In the clinical setting, skin of patients with post inflammatory hyperpigmentation, melasma and lentigines displayed decreased NNT expression levels, emphasizing the potential clinical relevance of this mechanism. Finally, to study the biological relevance of these findings in humans, GWAS analysis was performed, and identified four SNPs within the NNT gene, whose expression significantly correlated with human skin color.

In summary, these data reveal the existence of a redox dependent pigmentation mechanism, involving varied tyrosinase stabilization and skin pigmentation through melanosome maturation, which might be clinically modified by targeting NNT enzyme activity. In addition, to providing evidence of an MITF independent pathway towards regulation of human skin pigmentation, these results suggest that application of NNT modifying topical small molecules might be useful as a means of modulating skin pigmentation. Such approaches would definitely require careful attention to safety but may also offer unique opportunities with regard to skin cancer prevention strategies. Currently additional studies are needed to identify more specific compounds for modulating NNT enzyme activity. Finally, the exact mechanism(s) underlying many pigmentation disorders, such as post inflammatory hyper or hypopigmentation, melasma or lentigines, have not been fully elucidated. Correspondingly, currently available treatments also exhibit only limited efficacy. Identification of an MITF and UV independent mechanism of skin pigmentation that is potentially amenable to topical modulation may offer new prevention and treatment opportunities.

UV induces p53 that initiates the transcription of POMC in keratinocytes. Cleavage of POMC generates α-MSH that binds MC1R on adjacent melanocytes. This leads to cAMP elevation and activation of PKA. PKA phosphorylates CREB and inhibits the Salt Inducible Kinase (SIK) which led to the migration of cAMP-Regulated Transcriptional Co-activator (CRTC) to the nuclei. CRTC binds CREB and together induce the transcription of MITF. In turn, MITF activates the transcription of pigment genes (TYRP1, TYR and DCT/TRP2) leading to eumelanin production.

NNT transfers reducing equivalents from NADH to NADP, thereby generating reduced NADPH which, in turn is utilized to generate reduced glutathione in the mitochondrion. In melanocytes, depletion of NNT expression using siRNA or drug inhibitors regulates tyrosinase levels (and melanosome maturation) *via* changing cellular redox. This leads to tyrosinase stabilisation as well as other enzymes involved in melanin synthesis. Eumelanin levels increase during melanosome maturation [1–3].
